# Stress-enhanced cardiac lncRNA *Morrbid* protects hearts from acute myocardial infarction

**DOI:** 10.1172/jci.insight.165568

**Published:** 2023-08-22

**Authors:** Yang Yu, Haiqiong Yang, Qiuting Li, Nianhui Ding, Jiali Gao, Gan Qiao, Jianguo Feng, Xin Zhang, Jianming Wu, Yajun Yu, Xiangyu Zhou, Xiaobin Wang, Chunxiang Zhang

**Affiliations:** 1 Department of Cardiology, The Affiliated Hospital of Southwest Medical University,; 2 Key Laboratory of Medical Electrophysiology, Ministry of Education, Institute of Cardiovascular Research, Institute of Metabolic Diseases,; 3 School of Pharmacy, Southwest Medical University, Luzhou, China.; 4 Department of Anesthesiology, The Affiliated Hospital of Southwest Medical University, Luzhou, China .

**Keywords:** Cardiology, Cell Biology, Apoptosis, Cardiovascular disease, Noncoding RNAs

## Abstract

Myeloid RNA regulator of Bim-induced death (*Morrbid*) is a newly identified leukocyte-specific long noncoding RNA (lncRNA). However, the expression and biological functions of *Morrbid* in cardiomyocytes and heart disease are currently unclear. This study was meant to determine the role of cardiac *Morrbid* in acute myocardial infarction (AMI) and to identify the potential cellular and molecular mechanisms involved. We found that both human and mouse cardiomyocytes could express a significant amount of *Morrbid* and that its expression was increased in cardiomyocytes with hypoxia or oxidative stress as well as in mouse hearts with AMI. Overexpression of *Morrbid* reduced the myocardial infarct size and cardiac dysfunction, whereas the infarct size and cardiac dysfunction deteriorated in cardiomyocyte-specific *Morrbid*-KO (*Morrbid*^fl/fl^/Myh6-Cre) mice. We identified that *Morrbid* had a protective effect against hypoxia- or H_2_O_2_-induced apoptosis; this was also confirmed in vivo in mouse hearts after AMI. We further discovered that serpine1 was a direct target gene of *Morrbid* that was involved in the *Morrbid*-mediated protective effect on cardiomyocytes. In summary, we have found, for the first time to our knowledge, that the cardiac *Morrbid* is a stress-enhanced lncRNA that protects hearts from AMI via antiapoptosis through its target gene serpine1. *Morrbid* may be a novel promising therapeutic target for ischemic heart diseases such as AMI.

## Graphical abstract

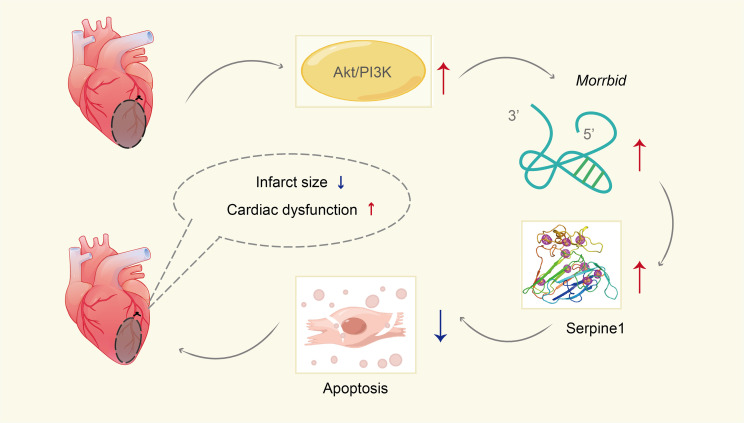

## Introduction

Despite the intensive modern treatments, the acute myocardial infarction (AMI) induced by serious hypoxia-ischemia is still one of the leading causes of death around the world due to our limited understanding of the critical molecular mechanisms that govern hypoxia-ischemia–mediated cardiac cell damage (1–3). Thus, it is urgent for us to discover novel molecular mechanisms and develop new therapies for AMI (3).

Long noncoding RNAs (lncRNAs) are defined as noncoding RNAs longer than ~200 nucleotides with strong biological functions. Recent studies have identified that a group of lncRNAs is related to AMI that might represent a novel class of diagnostic biomarkers and therapeutic targets (4, 5). Myeloid RNA regulator of Bim-induced death (*Morrbid*) is a leukocyte-specific lncRNA identified in 2016 that is conserved between mice and humans and is a key control factor for the leukocyte lifespan (6). Another study has reported that the leukocyte-specific *Morrbid* is associated with left ventricular hypertrophy (7).

To date, it is unclear whether or not the cardiomyocytes could express *Morrbid* and what the roles of *Morrbid* are in heart diseases such as AMI. To this end, we have identified that both human and mouse cardiac myocytes could express a significant amount of *Morrbid*. *Morrbid* expression is significantly increased in cardiomyocytes in response to hypoxia stress or oxidative stress and is significantly increased in mouse hearts after AMI. The current study is, thus, intended to determine the role of cardiac myocyte *Morrbid* in AMI and to identify the potential cellular and molecular mechanisms involved.

## Results

### Morrbid is expressed in cardiac cells, and its expression is increased in injured cardiomyocytes and in mouse hearts with AMI.

**Figure 1:**
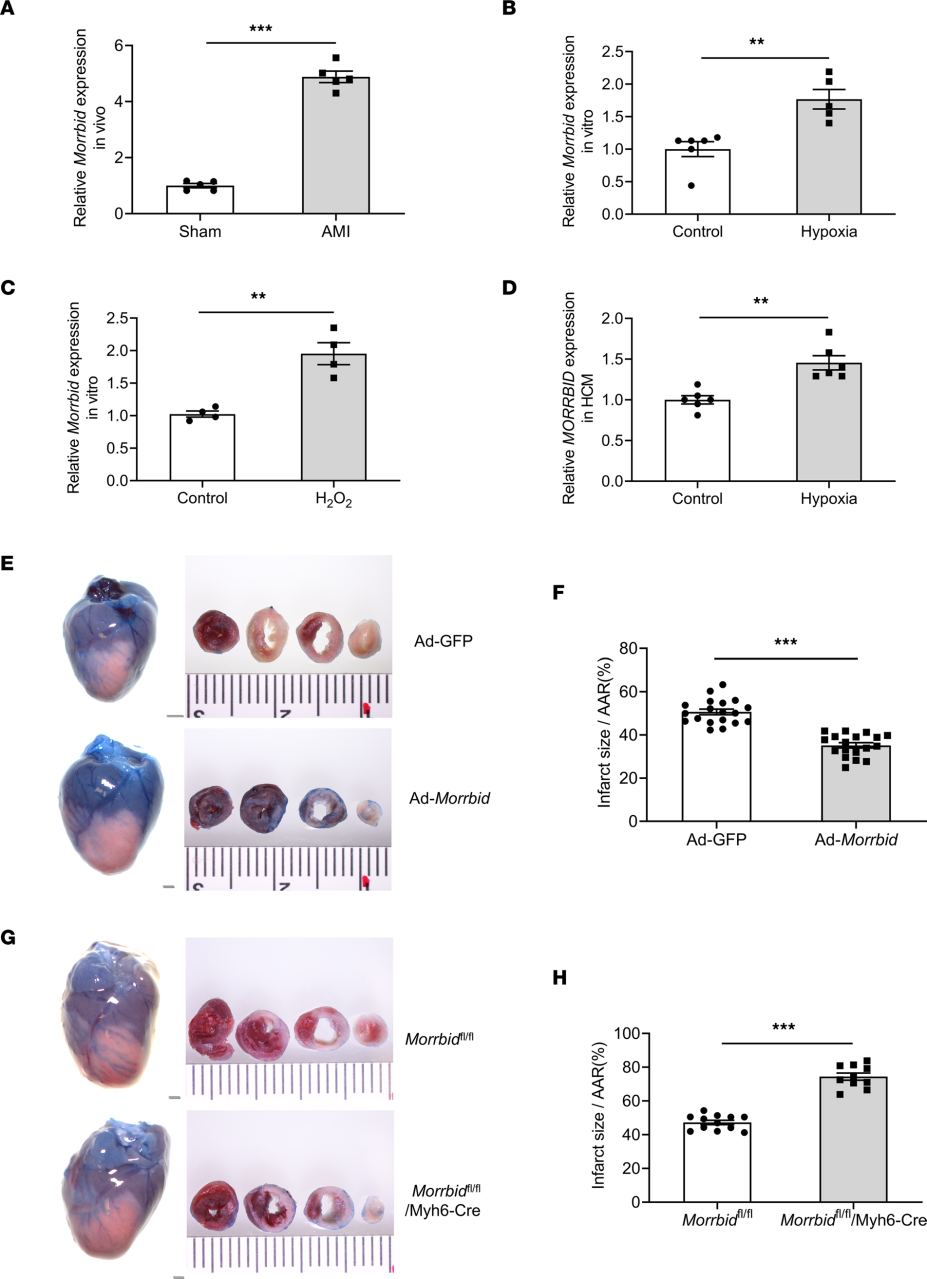
The expression of *Morrbid* in cardiomyocytes and in hearts and its effect on myocardial infarct size. (**A**) The expression of *Morrbid* in sham-operated mouse hearts and in mouse hearts at 24 hours after AMI (*P* < 0.001; Sham, *n* = 5; AMI, *n* = 5). (**B**) The expression of *Morrbid* in cultured neonatal mouse cardiomyocytes (NMCM) with or without hypoxia treatment for 24 hours (*P* = 0.0025; Control, *n* = 6; Hypoxia, *n* = 5). (**C**) The expression of *Morrbid* in cultured NMCM treated with vehicle or H_2_O_2_ (50 μM) for 12 hours (*P* = 0.0019; Control, *n* = 4; H_2_O_2_, *n* = 4). (**D**) The expression of *Morrbid* in cultured human cardiomyocytes (HCMs) with or without hypoxia treatment for 48 hours (*P* = 0.0011; Control, *n* = 6; Hypoxia, *n* = 6). (**F**) Ad-*Morrbid* (2.1 × 10^8^ pfu) reduced myocardial infarct size compared with Ad-GFP–treated control mouse hearts (*P* < 0.001; Ad-GFP, *n* = 19; Ad-*Morrbid*, *n* = 18). (**H**) The myocardial infarct size was increased in *Morrbid*^fl/fl^/Myh6-Cre mice compared with *Morrbid*^fl/fl^ mice (*P* < 0.001; *Morrbid*^fl/fl^, *n* = 12; *Morrbid*^fl/fl^/Myh6-Cre, *n* = 10). (**E** and **G**) Representative TTC- and Evans blue–stained heart slices. Scale bars: 1 mm. *P < 0.05; **P < 0.01; ***P < 0.001 by unpaired 2-tailed Student’s *t* tests.

Via quantitative PCR (qPCR) analysis, we identified that both mouse cardiomyocytes and mouse heart tissues had a significant amount of *Morrbid*. Compared with that in the sham-operated group, the expression of *Morrbid* in mouse hearts 24 hours after AMI was significantly upregulated (Figure 1A). In addition, the expression of *Morrbid* in the infarcted area was increased, compared with that in the noninfarcted area in the same mouse hearts at 24 hours after AMI (Supplemental Figure 1 and Supplemental Table 1; supplemental material available online with this article; https://doi.org/10.1172/jci.insight.165568DS1). The time-course changes of *Morrbid* in the infarcted mouse hearts within 24 hours after AMI was shown in Supplemental Figure 2.

In cultured mouse cardiomyocytes, the expression of *Morrbid* after hypoxia for 24 hours was increased, compared with the nonhypoxia control group (Figure 1B). Under treatment with H_2_O_2_ (50 μM), another injury stimulus under AMI conditions, the expression of *Morrbid* in mouse cardiomyocytes was also increased (Figure 1C). To improve the translational feature of our study, we determined the *Morrbid* expression levels in human cardiomyocytes (HCMs) with and without hypoxia stress. We found that HCMs also had a significant amount of *Morrbid*, similar to mouse cardiomyocytes. Interestingly, as shown in Figure 1D, the expression of *Morrbid* was also upregulated in cultured HCMs treated with hypoxia. Thus, *Morrbid* could be a stress-enhanced lncRNA in both mouse cardiomyocytes and HCMs — at least regarding hypoxia stress and oxidative stress, the 2 well-established injury stimuli in AMI.

To date, the expression control of *Morrbid* in cardiomyocytes is completely unknown. PI3-kinase/Akt is a well-known signaling pathway that is activated in cardiomyocytes after AMI and hypoxia. To test the potential involvement of PI3-kinase/Akt in the expression control of *Morrbid*, PI3-kinase/Akt in cultured cardiomyocytes was inhibited by its inhibitor LY294002. As shown in Supplemental Figure 3, the PI3-kinase/Akt inhibitor LY294002 could indeed inhibit the expression of *Morrbid* in cultured mouse cardiomyocytes.

### The effect of Morrbid on infarct size in mice with AMI.

In order to determine the effect of *Morrbid* on the infarct size after AMI, the mice were divided into 2 groups: the *Morrbid*-overexpressed group via adenovirus-expressing *Morrbid* (Ad-*Morrbid*) and the control group via Ad-GFP (Figure 1F). Ad-*Morrbid* or Ad-GFP was delivered into the mice hearts using the local delivery method at a dose of 2.1 × 10^8^ pfu/mouse to modulate the expression of *Morrbid* at 2 days before AMI. The results show that myocardial infarct size in the *Morrbid*-overexpressed group was significantly reduced compared with that in the Ad-GFP–treated control group (Figure 1F).

To further confirm the role of *Morrbid* in myocardial infarct size, a loss-of-function approach via cardiomyocyte-specific *Morrbid*-KO (*Morrbid*^fl/fl^/Myh6-Cre) mice was applied. As shown in Figure 1H, the myocardial infarct size in cardiomyocyte-specific *Morrbid*-KO (*Morrbid*^fl/fl^/Myh6-Cre) mice was significantly increased, compared with the control *Morrbid*^fl/fl^ group. Representative triphenyl tetrazolium chloride–stained (TTC-stained) and Evans blue–stained heart slices from these mice are shown in Figure 1, E and G.

### The effect of Morrbid on cardiac function in mice after AMI.

**Figure 2:**
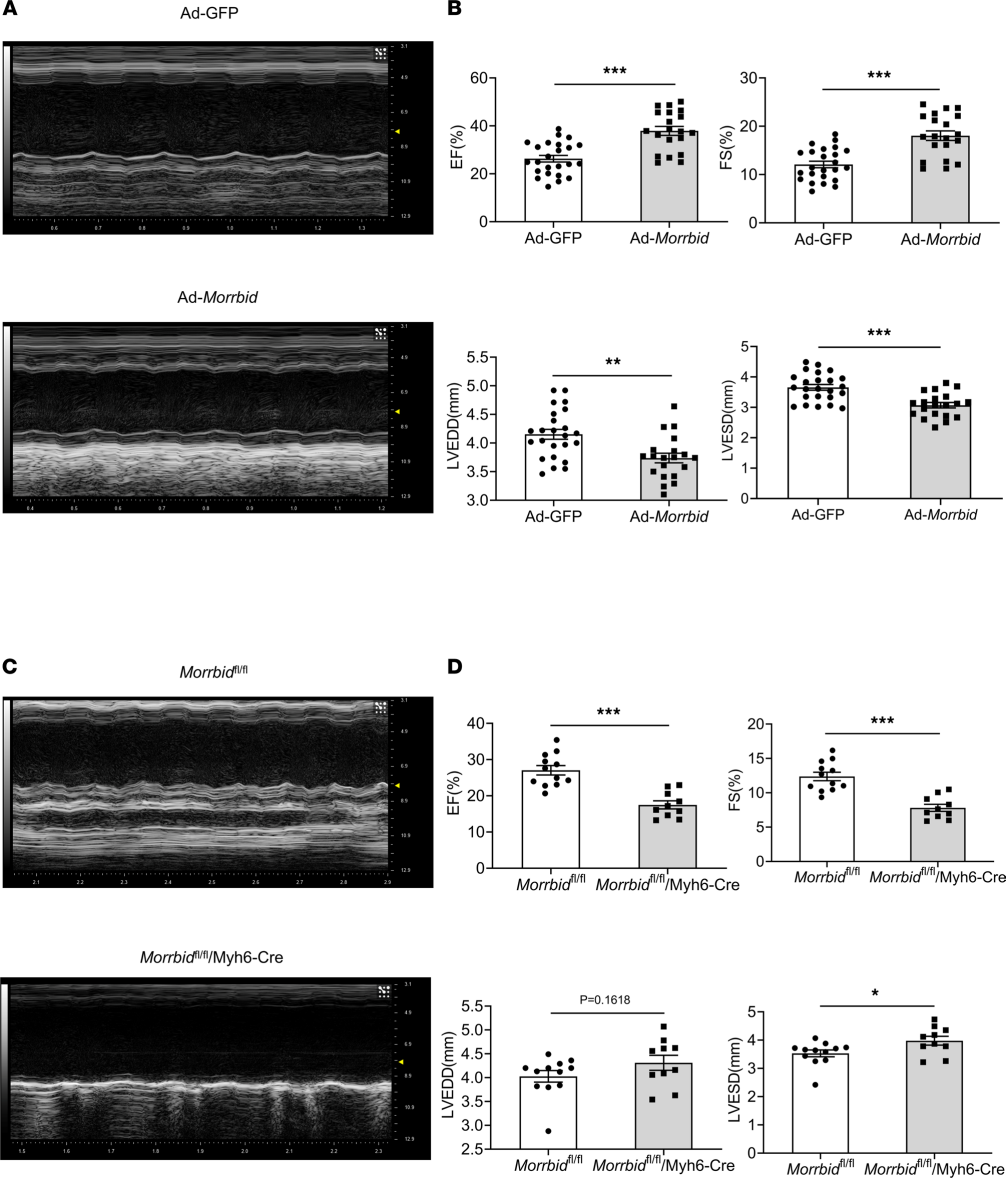
The effect of *Morrbid* on cardiac function at 24 hours after AMI. (**A** and **C**) Representative echocardiographic images obtained from 4 groups of mice. (**B**) Echocardiographic measurements for Ad-*Morrbid*–treated mouse hearts and for Ad-GFP–treated control mouse hearts: EF (*P* < 0.001), FS (*P* < 0.001), LVEDD (*P* = 0.0015), LVESD (*P* < 0.001). (**D**) Echocardiographic measurements for *Morrbid*-KO mice (*Morrbid*^fl/fl^/Myh6-Cre) and their control mice (*Morrbid*^fl/fl^): EF (*P* < 0.001), FS (*P* < 0.001), LVEDD (*P* = 0.1618), LVESD (*P* = 0.0332). Ad-GFP, *n* = 24; Ad-*Morrbid*, *n* = 20; *Morrbid*^fl/fl^, *n* = 12; *Morrbid*^fl/fl^/Myh6-Cre, *n* = 10. *P < 0.05; **P < 0.01; ***P < 0.001 by unpaired 2-tailed Student’s *t* tests.

Echocardiography was performed on mice to determine the effect of *Morrbid* on cardiac function after AMI. As shown in Figure 2, A and B, the Ad-*Morrbid*–treated group had better ejection fraction (EF) and fractional shortening (FS), as well as smaller left ventricular end-diastolic dimension (LVEDD)and left ventricular end-systolic dimension (LVESD), compared with the Ad-GFP–treated group. In contrast, cardiomyocyte-specific *Morrbid*-KO (*Morrbid*^fl/fl^/Myh6-Cre) mice had the worse EF and FS, and larger LVEDD and LVESD, compared with the control *Morrbid*^fl/fl^ group (Figure 2, C and D). Representative echocardiographic images obtained from 4 groups of mice were shown in Figure 2, A and C.

### The effect of Morrbid on cardiac cell apoptosis in mouse hearts at 24 hours after AMI.

**Figure 3:**
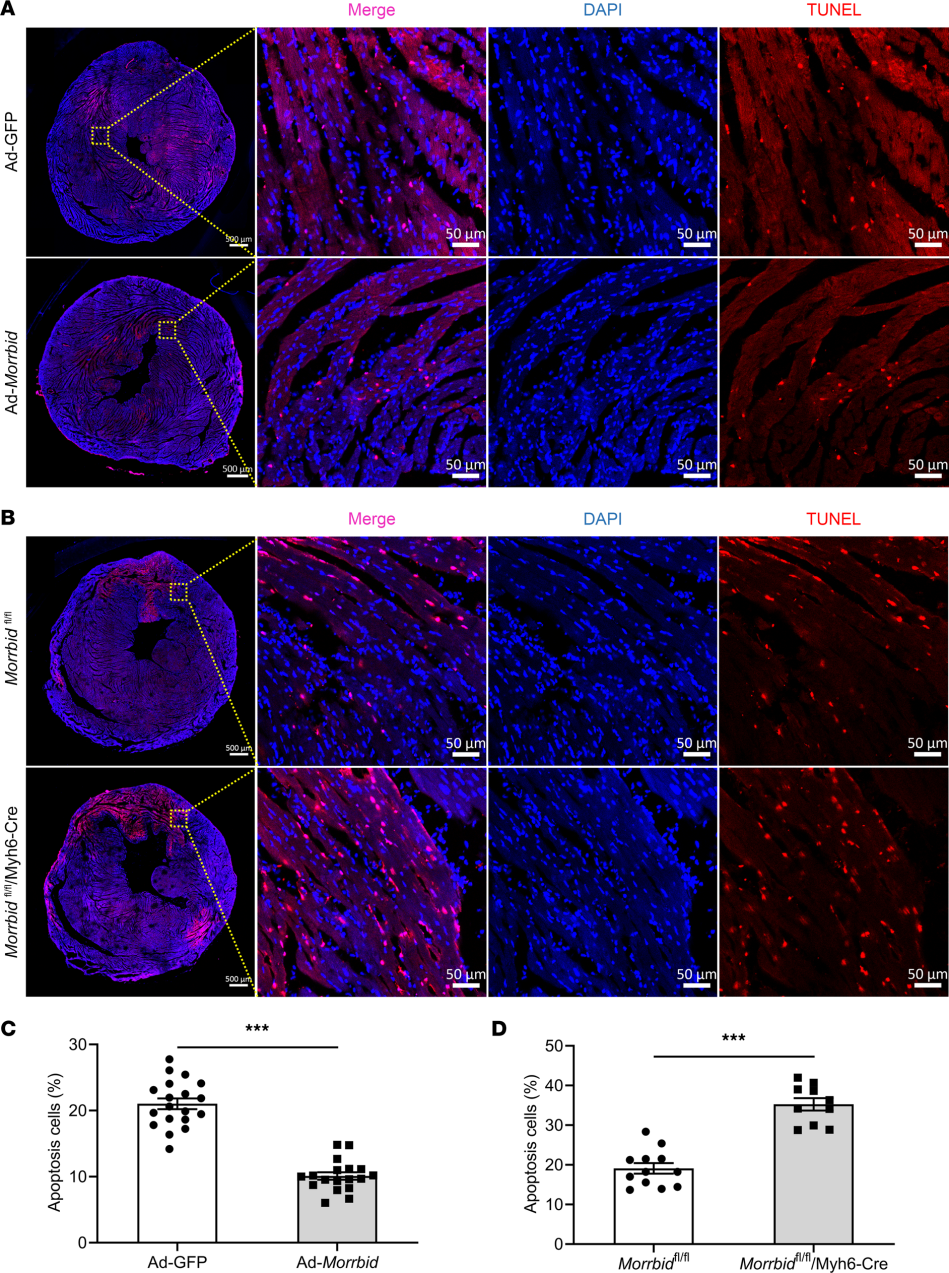
The effect of *Morrbid* on cell apoptosis in mouse hearts at 24 hours after AMI. (**A** and **B**) Representative TUNEL-stained photomicrographs of cardiac cells in heart sections from *Morrbid*^fl/fl^/Myh6-Cre mice, *Morrbid*^fl/fl^ mice, and WT mice treated with Ad-GFP or Ad-*Morrbid* at 24 hours after AMI. Scale bars: 50 μm or 500 μm. (**C**) Quantitative analysis of the apoptotic cells in heart sections from mice treated with Ad-GFP or Ad-*Morrbid* at 24 hours after AMI (*P* < 0.001; Ad-GFP, *n* = 19; Ad-*Morrbid*, *n* = 18). (**D**) Quantitative analysis of the apoptotic cells in heart sections from *Morrbid*^fl/fl^/Myh6-Cre mice or *Morrbid*^fl/fl^ mice (*P* < 0.001; *Morrbid*^fl/fl^, *n* = 12; *Morrbid*^fl/fl^/Myh6-Cre, *n* = 10). Red, apoptotic cell; blue, the cell nucleus stained by DAPI. ***P < 0.001 by unpaired 2-tailed Student’s *t* tests.

In order to determine the potential cellular mechanism involved in *Morrbid*-mediated cardiac protection in vivo, apoptosis was determined in heart sections from these 4 groups of mice by immunofluorescence with TUNEL staining. As shown in Figure 3C, cardiac cell apoptosis was decreased in Ad-*Morrbid*–treated mice. In contrast, cardiac cell apoptosis was significantly increased in *Morrbid*^fl/fl^/Myh6-Cre mice with the cardiomyocyte-specific *Morrbid* KO (Figure 3D). Representative TUNEL-stained photomicrographs of cardiac cells in heart sections from these groups of mice were shown in Figure 3, A and B.

### The effect of Morrbid on hypoxia-induced cardiac cell apoptosis in vitro.

**Figure 4:**
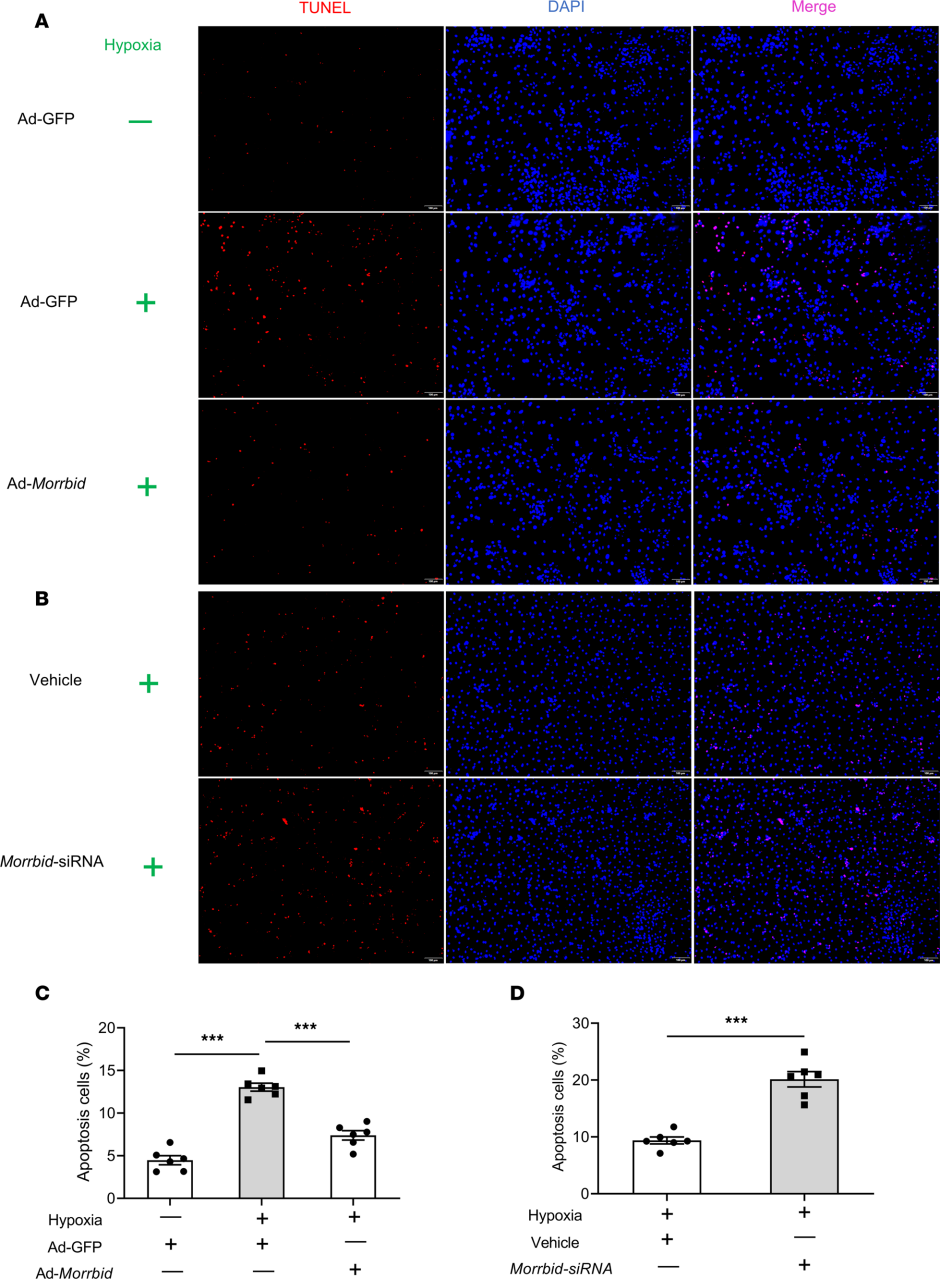
The effect of *Morrbid* on hypoxia-induced cardiac cell apoptosis in vitro. Cultured cardiomyocyte apoptosis was induced by hypoxia for 24 hours in a serum-free and low-glucose medium. Cell apoptosis was determined by TUNEL staining. (**A** and **B**) Representative TUNEL-stained photomicrographs from cardiomyocytes treated with Ad-GFP, Ad-*Morrbid*, vehicle, or *Morrbid* siRNA. Scale bars: 100 μm. (**C**) Hypoxia resulted in an increase in apoptosis (*P* < 0.001; Ad-GFP without hypoxia, *n* = 6; Ad-GFP hypoxia, *n* = 6), and the increased apoptosis was inhibited after treatment with Ad-*Morrbid* (*P* < 0.001; Ad-*Morrbid* with hypoxia, *n* = 6; Ad-GFP with hypoxia, *n* = 6). (**D**) Cardiomyocyte apoptosis was exacerbated after treatment with *Morrbid* siRNA (*P* < 0.001; Vehicle, *n* = 6; *Morrbid* siRNA, *n* = 6). Red, apoptotic cell; blue, the cell nucleus stained by DAPI. ***P < 0.001 by 1-way ANOVA with Sidak’s post hoc correction for multiple comparisons (**C**) or unpaired 2-tailed Student’s *t* tests (**D**).

To further confirm the cellular mechanism involved in the *Morrbid*-mediated effect on cardiac protection, a cell hypoxia model was applied, in which the cultured neonatal mouse cardiomyocyte apoptosis was induced by hypoxia for 24 hours in a serum-free and low-glucose medium. As shown in Figure 4C, hypoxia resulted in an increase in apoptosis. Neonatal mouse cardiac myocyte apoptosis was inhibited after treatment with Ad-*Morrbid* compared with the control Ad-GFP group. We also used the *Morrbid* siRNA to inhibit the expression of *Morrbid* and found that neonatal mouse cardiac myocyte apoptosis was exacerbated after treatment with *Morrbid* siRNA compared with the control group (Figure 4D). Representative TUNEL-stained photomicrographs of cardiomyocytes from different groups were shown in Figure 4, A and B. To verify the antiapoptotic effect of *Morrbid* in adult cardiomyocytes, we isolated mouse cardiomyocytes from adult WT mice (*Morrbid*^fl/fl^ mice) and adult cardiomyocyte-specific *Morrbid*-KO mice (*Morrbid*^fl/fl^/Myh6-Cre mice). We found that *Morrbid* was also a stress-enhanced lncRNA in adult cardiomyocytes (Supplemental Figure 4). The cell apoptosis of these adult *Morrbid*-KO mouse cardiomyocytes was exacerbated compared with the adult mouse cardiomyocytes from the WT control group (Supplemental Figure 5).

### Serpine1 is a target gene of Morrbid.

**Figure 5:**
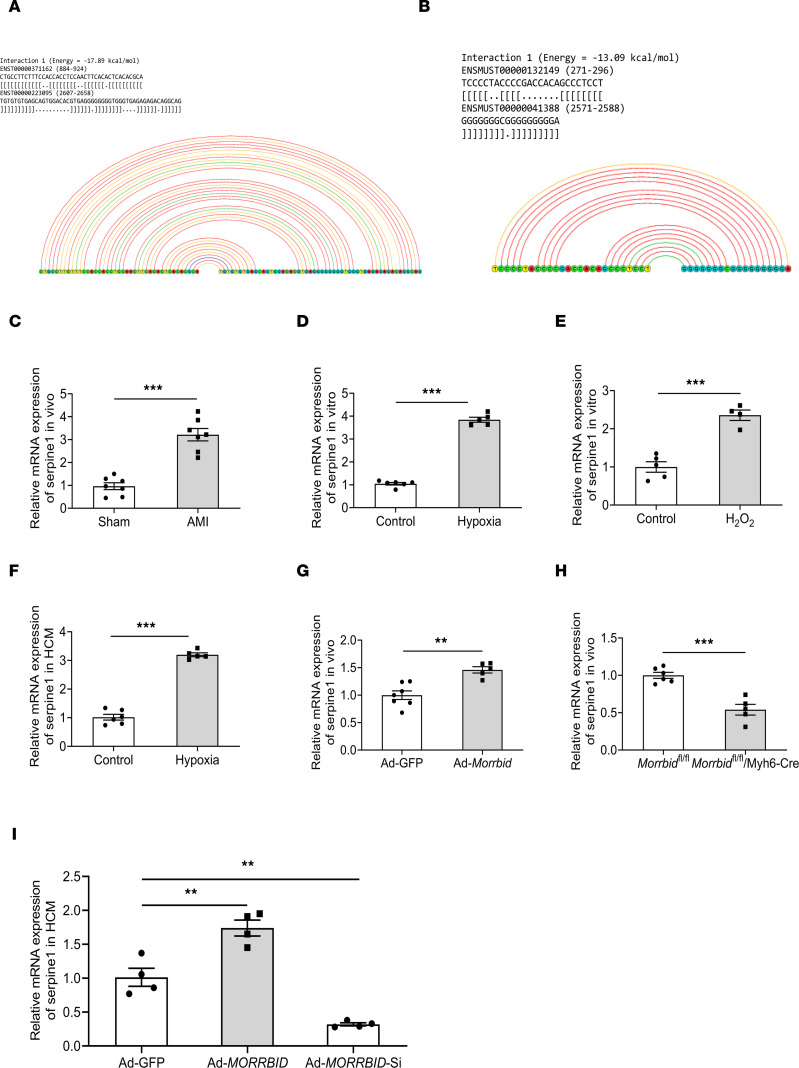
The expression relationship between *Morrbid* and serpine1 mRNA. (**A** and **B**) Computational analysis indicates that serpine1 mRNA has binding sites with both human and mouse *Morrbid*. (**C**) The expression of serpine1 mRNA in mouse hearts at 24 hours after AMI was increased (*P* < 0.001; Sham, *n* = 7; AMI, *n* = 7). (**D**) The expression of serpine1 mRNA in cultured NMCM with hypoxia for 24 hours was increased (*P* < 0.001; Control, *n* = 6; Hypoxia, *n* = 5). (**E**) The expression of serpine1 mRNA in cultured NMCM treated with H_2_O_2_ (50 μM) for 12 hours was increased (*P* < 0.001; Control, *n* = 5; H_2_O_2_, *n* = 4). (**F**) The expression of serpine1 mRNA in cultured HCMs with hypoxia for 48 hours was increased (*P* < 0.001; Control, *n* = 6; Hypoxia, *n* = 5). (**G**) The expression of serpine1 mRNA in the Ad-*Morrbid*–treated mouse hearts was increased (*P* = 0.0014, Ad-GFP, *n* = 7; Ad-*Morrbid*, *n* = 5). (**H**) The expression of serpine1 mRNA in cardiomyocyte-specific *Morrbid*-KO mouse hearts was decreased (*P* < 0.001, *Morrbid*^fl/fl^, *n* = 6; *Morrbid*^fl/fl^/Myh6-Cre, *n* = 5). (**I**) The expression of serpine1 mRNA in the Ad-*Morrbid*–treated HCMs was increased (*P* = 0.0015, Ad-GFP, *n* = 4; Ad-*Morrbid*, *n* = 4), whereas Ad-*Morrbid* siRNA–treated (Ad-*Morrbid*-Si–treated) HCMs decreased the expression of serpine1mRNA (*P* = 0.0021, Ad-GFP, *n* = 4; Ad-*Morrbid* siRNA, *n* = 4). Thus, *Morrbid* positively regulated the expression of serpine1 mRNA. **P < 0.01; ***P < 0.001 by unpaired 2-tailed Student’s *t* tests (**C**–**H**) or 1-way ANOVA with Sidak’s post hoc correction for multiple comparisons (**I**).

Computational analysis indicates that mRNA of serpine1 has binding sites both in human and mouse *Morrbid* (Figure 5, A and B), suggesting that serpine1 might be a target gene of *Morrbid*. To test this, we first determined serpine1 mRNA expression in the different kinds of samples that we collected. As shown in Figure 5C, serpine1 mRNA expression was increased in mouse AMI samples. Both hypoxia and H_2_O_2_ stimuli increased the serpine1 mRNA expression in cultured mouse cardiomyocytes (Figure 5, D and E). In addition, in cultured HCMs, hypoxia also upregulated the expression of serpine1 mRNA (Figure 5F).

To confirm the relationship between *Morrbid* and serpine1 mRNA, we tested the *Morrbid*-overexpressed and *Morrbid*-deficient (*Morrbid*^fl/fl^/Myh6-Cre) mouse samples. As shown in Figure 5G, in *Morrbid*-overexpressed hearts via Ad-*Morrbid*, the serpine1 mRNA expression was increased. In contrast, in *Morrbid*-deficient mouse samples, the expression of serpine1 mRNA was decreased (Figure 5H). In cultured HCMs, we also found that serpine1 mRNA expression was increased by *Morrbid* overexpression but decreased by *Morrbid* knockdown (Figure 5I).

**Figure 6:**
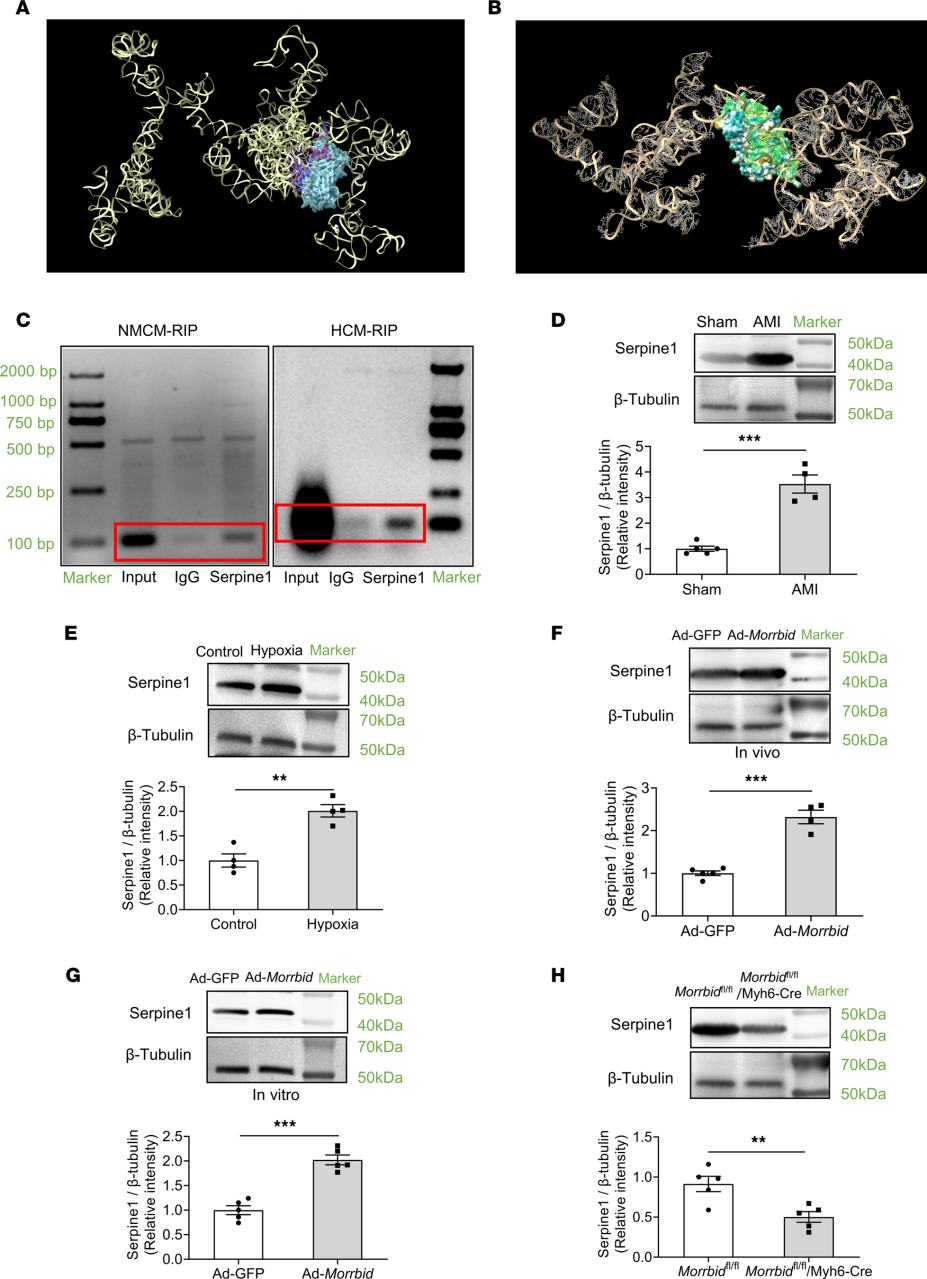
The expression relationship between *Morrbid* and serpine1 protein. (**A** and **B**) Computational analysis indicates that Serpine1 protein could have the direct interaction with both human and mouse *Morrbid*. (**C**) The RNA immunoprecipitation (RIP) results of mouse cardiomyocyte (NMCM) and human cardiomyocyte (HCM) revealed that *Morrbid* could directly bind to the serpine1 protein. (**D**) The expression of serpine1 protein in mouse hearts at 24 hours after AMI was increased (*P* < 0.001; Sham, *n* = 5; AMI, *n* = 4). (**E**) The expression of serpine1 protein in cultured NMCM treated with hypoxia for 24 hours was increased (*P* = 0.0015; Control, *n* = 4; Hypoxia, *n* = 4). (**F**) The expression of serpine1 protein in mouse hearts treated with Ad-*Morrbid* was increased (*P* < 0.001; Ad-GFP, *n* = 5; Ad-*Morrbid*, *n* = 4). (**G**) The expression of serpine1 protein in cultured NMCM treated with Ad-*Morrbid* was increased (*P* < 0.001; Ad-GFP, *n* = 5; Ad-*Morrbid*, *n* = 5). (**H**) The expression levels of serpine1 in cardiomyocyte-specific *Morrbid*-KO mouse hearts was decreased (*P* = 0.0076; *Morrbid*^fl/fl^, *n* = 5; *Morrbid*^fl/fl^/Myh6-Cre, *n* = 5). Thus, *Morrbid* has a direct interaction with its target gene serpine1 protein and positively regulates the expression of serpine1 protein. *P < 0.05; **P < 0.01; ***P < 0.001 by unpaired 2-tailed Student’s *t* tests.

To test the relationship between *Morrbid* and serpine1 protein, the AlphaFold2 and RoseTTAFold (a 3-track network software tool for protein structure prediction) were used to create the 3D protein structures of human and mouse protein serpine1 by using the Colab server (https://colab.research.google.com/github/sokrypton/ColabFold/blob/main/AlphaFold2.ipynb). The 3D structure of *Morrbid* was modeled by 3dRNA (v2.0) (http://biophy.hust.edu.cn/3dRNA/) with the input of a selected sequence of *Morrbid* by RNAfold (8, 9). To identify the mechanism of *Morrbid* binding with serpine1 protein, we performed serpine1:*Morrbid* docking model with the HDOCK server to identify the residues involved in the serpine1:*Morrbid* complex (10). The complex of serpine1:*Morrbid* was visualized and analyzed by UCSC Chimerax and BIOVIA Discovery Studio Visualizer. Based on the bioinformatics analysis, both human and mouse *Morrbid* might have direct interaction with its target gene serpine1 at the protein level (Figure 6, A and B). The detailed potential binding sites of human *Morrbid* with human serpine1 protein are displayed in Supplemental Figure 6A, and the binding sites of mouse *Morrbid* with mouse serpine1 protein are displayed in Supplemental Figure 6B. RNA immunoprecipitation (RIP) was then used to verify the results of computational analysis. As shown in Figure 6C, *Morrbid* could indeed directly bind to the serpine1 protein in both mouse cardiomyocytes and HCMs.

We also determined the protein levels of serpine1 via Western blot. As shown in Figure 6D, serpine1 protein level was increased in mouse hearts after AMI. In cultured mouse cardiomyocytes, hypoxia could upregulate the expression of serpine1 protein (Figure 6E). In *Morrbid*-overexpressed mouse hearts and *Morrbid*-overexpressed mouse cardiomyocytes via Ad-*Morrbid*, the serpine1 protein expression was increased (Figure 6, F and G), whereas in mouse hearts with *Morrbid* KO, the serpine1 protein expression was significantly decreased (Figure 6H).

### Serpine1 is involved in Morrbid-mediated antiapoptosis effect on cardiac myocytes.

**Figure 7:**
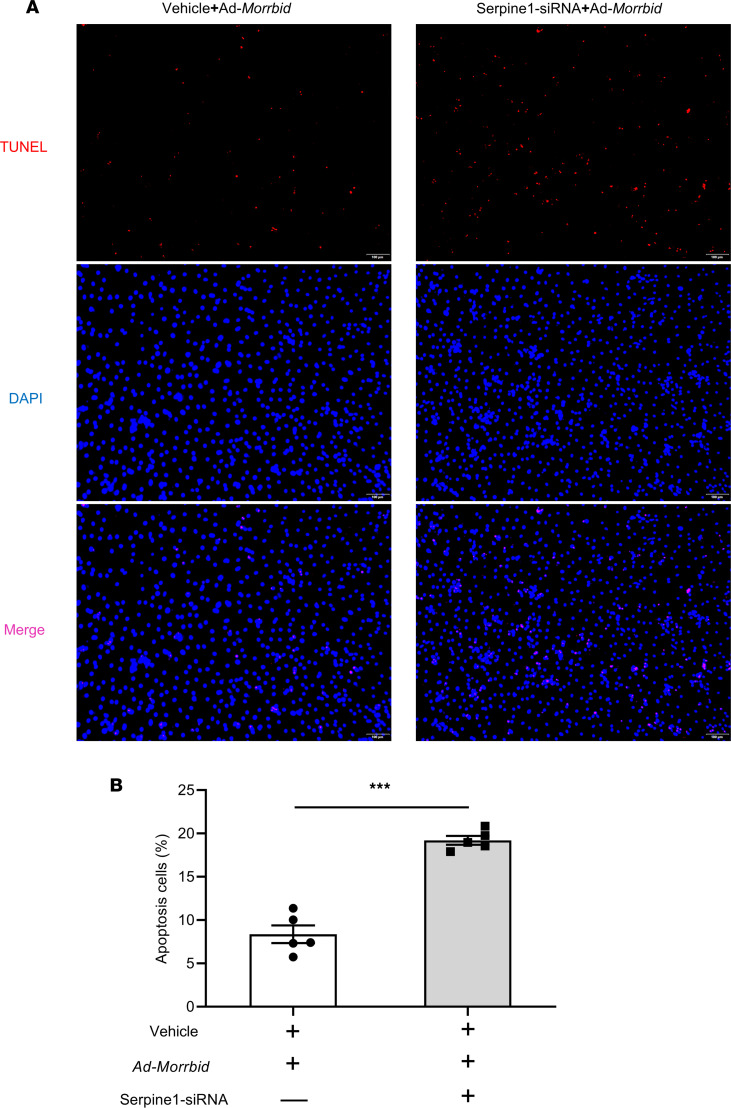
Serpine1 is involved in *Morrbid*-mediated antiapoptotic effect on cardiac myocytes. (**A**) Representative TUNEL-stained photomicrographs from Ad-*Morrbid*–treated cardiomyocytes with or without serpine1 deficiency (serpine1 siRNA). Scale bars: 100 μm. (**B**) Serpine1-siRNA could inhibit the protective effect of *Morrbid* on hypoxia-induced cardiomyocyte apoptosis (*P* < 0.001; Vehicle, *n* = 5; serpine1-siRNA, *n* = 5). ***P < 0.001 by unpaired 2-tailed Student’s *t* test.

In this experiment, cultured mouse cardiomyocytes were treated with either serpine1-siRNA (for serpine1 knockdown) or vehicle as the control. The *Morrbid* overexpression–mediated anti-apoptotic effects (via Ad-*Morrbid*) were determined in these cardiomyocytes after hypoxia. As shown in Figure 7, A and B, the protective effect of *Morrbid* on hypoxia-induced cardiac cell apoptosis was inhibited via serpine1 siRNA. Representative TUNEL-stained photomicrographs were shown in Figure 7A.

## Discussion

*Morrbid* is a recently identified leukocyte-specific/enriched lncRNA that has an antiapoptotic effect on leukocytes via its target gene Bim to control the leukocyte lifespan (6). In this study, we identified that both human and mouse cardiomyocytes could express a significant amount of *Morrbid*. For example, we found that, among the isolated RNAs from mouse cardiomyocytes, the CT level of *Morrbid* via qPCR was about 20.5, while β-actin mRNA and GAPDH mRNA — the 2 abundant mRNAs in cardiomyocytes — were about 16 and 14.8. In mouse hearts, we also found that *Morrbid* was highly expressed (CT levels: *Morrbid*, 23.5; β-actin, 16.2; GAPDH, 14.5).

One interesting discovery of this study is that we have found that cardiac *Morrbid* is a stress-enhanced lncRNA. Indeed, the results show that, in response to hypoxia stress or oxidative stress, the expression of *Morrbid* in cardiomyocytes was significantly increased. The effects of other stresses on the expression of cardiac cell *Morrbid* should be determined to further verify it as a stress-enhanced lncRNA. If it is a stress-enhanced lncRNA, *Morrbid* might be a novel biomarker for cell stress. Since hypoxia stress or oxidative stress are 2 well-established injury stresses in many diseases, including AMI, we hypothesized that, in hearts after AMI, the expression of *Morrbid* might be increased. Our result in the mouse model of AMI clearly shows that the expression of *Morrbid* was indeed significantly increased in the infarcted mouse hearts.

AMI is induced by either a complete stop or a serious reduction of coronary artery blood supply to heart cells and tissues. In response to the ischemic/hypoxia injury, many protein-coding genes and noncoding genes including noncoding RNAs inside cardiomyocytes are quickly changed (1–3). Some of these changes are responsible for the damage responses, including apoptosis and necrosis of the cardiomyocytes. In contrast, some of these changes are important endogenous protective mechanisms against AMI-induced cardiomyocyte death (3). To test the potential role of the stress-enhanced cardiac lncRNA in AMI, we first determined the effects of *Morrbid* on hypoxia and H_2_O_2_-induced cardiomyocyte apoptosis. We found that *Morrbid* overexpression could effectively inhibit both hypoxia and H_2_O_2_-induced cardiomyocyte apoptosis, whereas the hypoxia and H_2_O_2_-induced cardiomyocyte apoptosis was aggravated via *Morrbid* knockdown.

*Morrbid* is a leukocyte-enriched lncRNA that might play important roles in leukocyte functions (6), whereas inflammatory leukocytes play critical roles in AMI (11). Indeed, many leukocytes are infiltrated into the infarcted and ischemic hearts with biological roles in the whole progress of AMI. For the in vivo study, to avoid interference from leukocyte *Morrbid*, we first gave the local heart tissue injection of Ad-*Morrbid* at 2 days before AMI to overexpress *Morrbid* in cardiac cells, since most cell types in noninfarcted normal hearts are cardiomyocytes. We found that overexpression of *Morrbid* in cardiac tissues/cells inhibited the myocardial infarct size and cardiac dysfunction in the mouse model of AMI. To further provide strong evidence about the effect of cardiomyocyte *Morrbid* on AMI, we generated cardiomyocyte-specific *Morrbid*-KO mice. Clearly, in mice with cardiomyocyte-specific *Morrbid* deficiency, myocardial infarct size and cardiac dysfunction deteriorated after AMI. In addition, the cardiac cell apoptosis in mouse heart tissues was inhibited by *Morrbid* overexpression but was increased by *Morrbid* KO. Thus, the stress-enhanced cardiomyocyte *Morrbid* has a protective role in AMI-induced cardiac damage via its antiapoptotic effect on cardiomyocytes. Other cellular mechanisms involved in cardiomyocyte *Morrbid*–mediated protective effects on AMI should be determined in future studies.

It is well known that a lncRNA achieves its biological functions via its multiple target genes. To uncover the molecular mechanisms involved in the *Morrbid*-mediated effect on cardiomyocyte apoptosis, we first performed the bioinformatics analysis and found that *Morrbid* has binding sites on both serpine1 mRNA and serpine1 protein. Thus, serpine1 could be a target gene for *Morrbid*. Serpine1 is a known hypoxia-sensitive gene, as reported in a previous study on cancer cells (12). In the current study, we found that serpine1 expression was increased in hypoxia-treated mouse cardiomyocytes and HCMs and in mouse hearts after AMI. By using both the gain-of-function and loss-of-function approaches, we identified that *Morrbid* had a strong regulatory effect on the expression of serpine1 at both mRNA and protein levels in cardiomyocytes. The regulatory effect of *Morrbid* on serpine1 expression was further verified in vivo in mouse hearts. In addition, the direct binding ability between *Morrbid* and serpine1 protein was identified by RIP assay. Finally, we found that serpine1 was involved in a *Morrbid*–mediated protective effect on cardiomyocyte apoptosis. Clearly, serpine1 is a direct functional target gene in cardiomyocytes that is related to *Morrbid*–mediated cardiac cell protection from ischemic damage. Other target genes of *Morrbid* in cardiomyocytes should be identified in future studies.

One remaining question is how *Morrbid* expression is regulated in cardiomyocytes. We found that *Morrbid* expression was increased in cardiomyocytes with hypoxia and oxidative stresses, while the PI3-kinase/Akt was a key activated signaling pathway in these stressed cardiomyocytes. We thus tested whether the PI3-kinase/Akt signaling pathway could regulate the expression of *Morrbid*. The result showed that PI3-kinase/Akt was indeed able to regulate the *Morrbid* expression in cardiomyocytes.

There are some limitations in this study. First, we only performed the *Morrbid* expression experiment and apoptosis experiment in adult mouse cardiomyocytes. Additional experiments should be performed in adult mouse cardiomyocytes to further verify the molecular mechanism in a *Morrbid*-mediated cellular effect on cardiomyocytes. Second, although we found that *Morrbid* could control the expression of its target gene serpine1 at both mRNA and protein levels, the detailed regulatory mechanisms still need to be investigated. Third, although we found that the expression of *Morrbid* was also increased in female mouse hearts after AMI, in this study, we focused on male mice. To this end, we don’t know whether female hormone, a well-known factor to protect the heart, could affect the *Morrbid*-mediated effect on AMI. This was a weakness of our study. The role of *Morrbid* in AMI should also be determined in female mice.

In summary, in this study, we have identified that both human and mouse cardiomyocytes could express a significant amount of *Morrbid*, which is increased in cardiomyocytes in response to hypoxia stress or oxidative stress and in mouse hearts in response to AMI. The stress-enhanced cardiac cell *Morrbid* plays a critical protective role in AMI via antiapoptosis through its target gene serpine1. *Morrbid* might be a novel promising therapeutic target for ischemic heart diseases such as AMI.

## Methods

### AMI animal model.

AMI was induced in 10-week-old male C57BL/6 mice by ligation of the left anterior descending artery (LAD) (13). In brief, under general anesthesia with isoflurane (2%), the mice were fixed in the supine position on a warm operating plate. The hair on the left chest was shaved, and treatment with hair-removal cream followed. The operation site was disinfected using Povidone-Iodine Prep Pad (Dynarex), followed by wiping with 70% ethanol. The mouse heart was exposed via a small left thoracotomy on the third intercostal space. The pericardial sac was cut open, and the LAD was ligated at the level of about 3 mm below the left atrial appendix with a 7.0 silk suture. The success of the LAD ligation was confirmed by identifying the myocardium distal to the ligation site turning pale. We confirmed that the area of paleness (the myocardial area at risk [AAR]) was comparable among the groups. The comparable myocardial ischemic AAR among groups after LAD ligation were also confirmed by the Evans blue dye method (14). Then, the opened intercostal space was closed, followed by closure of the skin suture and manual evacuation of pneumothoraces. Sham operation was performed in the same manner, except for the LAD ligation.

### Measurement of infarct size.

Myocardial infarct size was determined by pathological staining of TTC as described in our previous study (15). In brief, at the end of the experiments, mice were anesthetized and 0.4 mL of 1% Evans blue dye was injected into the vena jugular to define the area that was not supplied by LAD. The myocardial ischemic AAR was identified as the region lacking blue staining. The ventricles of the hearts were sliced transversely into 2 mm–thick slices. The slices were incubated in 1% TTC at 37°C for 10 minutes to identify the noninfarcted and infarcted areas. TTC staining was displayed as red. The infarcted area was defined as TTC-unstained area (white). Infarct size was expressed as a percentage of the AAR (15). The infarcted heart tissues were also confirmed by H&E staining. The representative H&E staining of heart sections from different groups of mice were shown in Supplemental Figure 7.

### Transthoracic echocardiographic studies.

At 24 hours after AMI, transthoracic echocardiographic studies were performed under light anesthesia using a Vevo2100 ultrasound machine (Visual Sonics Inc.) equipped with a 30 MHz probe. Mice were anesthetized with isoflurane (1%) and subjected to echocardiography (16, 17). The LVESD and LVEDD were measured from the left ventricular M-mode recording. Percent EF and percent FS of the left ventricle (LV) were autocalculated by the echomachine.

### Generation of the cardiomyocyte-specific Morrbid-KO mice.

To generate *Morrbid*^fl/fl^ mice on a C57BL/6 background, we designed the Cas9/guide RNA (gRNA) target sequences to the regions upstream of exon1 and Intron1. The targeting construct consisted of 1.3 kb arms of homologous genomic sequence immediately upstream (5′) of exon 1 and Intron1, flanked by 2 loxP sites (Supplemental Figure 8A). Cas9 mRNA and sgRNAs were transcribed with T7 RNA polymerase in vitro. Cas9 mRNA, sgRNAs, and donor vector were mixed at different concentrations and coinjected into the cytoplasm of fertilized eggs at the 1-cell stage. The genotypes for *Morrbid*^fl/fl^ mice were validated by PCR amplification, direct sequencing, and Southern blot analysis, with the probes indicated (Supplemental Figure 8B). By crossing *Morrbid*^fl/fl^ and LysM-Cre mice that express Cre-recombinase in the cardiomyocyte lineage, we have been able to obtain cardiomyocyte-specific *Morrbid*-KO (Myh6-cre/*Morrbid*^fl/fl^) mice (Supplemental Figure 8C).

### Construction of the adenovirus-expressing Morrbid, Morrbid siRNA, or control adenovirus-expressing GFP.

The Ad-*Morrbid*, *Morrbid* siRNA (Ad-*Morrbid* siRNA), or control Ad-GFP was generated using the Adeno-5 Adenovirus Expression System (Applied Biological Materials Inc [Abm]) according to the manufacturer’s protocols. These adenoviruses were purified by cesium chloride gradient ultracentrifugation and titrated using median tissue culture infectious dose (TCID_50_).

### Adenovirus-mediated Morrbid gene transfer by intramyocardial injection.

Ad-*Morrbid* or Ad-GFP was delivered into the mouse hearts within the LAD-supplied area at 2 days before AMI with a dose of 2.1 × 10^8^ pfu/mouse as described (17). Briefly, under general anesthesia with isoflurane (2%), the heart was exposed via a small left thoracotomy on the third intercostal space. The LAD was identified, and 7 μL of adenovirus was injected in both sides of the LAD and left ventricular apex with an insulin syringe (BD SafetyGlide Insulin 0.25 mm × 6 mm). The needle formed an angle of 15° with the LV wall, and the penetration depth was about 0.5–0.8 mm when injected into the myocardium. Then, the opened intercostal space was closed, followed by closure of the skin suture and manual evacuation of pneumothoraces.

### Detection of apoptosis.

Cultured cardiac myocyte apoptosis and cell apoptosis in heart sections were measured by TUNEL staining as described previously (18, 19). Briefly, cardiac myocytes cultured on coverslips in 24-well plates were fixed in 4% paraformaldehyde. The number of TUNEL^+^ cells and the total cells were counted under a fluorescence microscope. In heart tissue, TUNEL staining was performed in frozen heart sections (10 μM) using the in situ cell death detection kit (Sigma-Aldrich). The numbers of TUNEL^+^ cells and the total cells in heart sections were counted under a fluorescence microscope. TUNEL-stained photomicrographs from cardiomyocytes treated with TMR-dUTP served as negative control staining, and normal TUNEL staining served as positive control staining (Supplemental Figure 7).

### Cardiac myocyte culture.

Primary culture of neonatal mouse cardiomyocytes (NMCM) was performed as described (20). In brief, hearts from 1- to 3-day-old C57BL/6 mice were placed in ice-cold 1× PBS (without Ca^2+^ or Mg^2+^ [CellGro]) supplemented with 20 mM 2,3-Butanedione 2-monoxime (BDM) (MilliporeSigma); after repeated rinsing, the lung tissue and larger vessels (as well as atria, if desired) were cut off, and the hearts were minced with scissors into small pieces (approximately 0.5–1 mm^3^ or smaller). Then, the minced hearts were transferred into a conical tube containing 10 mL of the isolation medium (on ice) and incubated with gentle agitation at 4°C overnight. The supernatant was removed, and 5 mL of digestion medium and 5 mL of L-15 (CellGro) supplemented with 20 mM BDM were added to tissue fragments and incubated at 37°C with gentle agitation for 20–30 minutes. Supernatant containing suspended cells was transferred into a fresh conical tube through a sterile cell strainer (40-100 μm nylon mesh; Thermo Fisher Scientific). Isolated cardiomyocytes were obtained by 5 minutes of centrifugation at approximately 100*g* at 4°C and incubated for 1–3 hours in a cell culture incubator to further remove any fibroblasts and endothelial cells. The cardiomyocytes were placed into collagen-coated cell culture dishes and left undisturbed for 12–18 hours to allow for adherence and spreading.

### Isolation and culture of cardiac myocytes from adult mouse heart.

Media and buffers were prepared as described (21). The EDTA buffer, perfusion buffer, and collagenase buffer were apportioned into sterile 10 mL syringes with 27G hypodermic needles. C57BL/6J or *Morrbid*^fl/fl^/Myh6-Cre mice aged 8–12 weeks were anesthetized, and the chest was opened to expose the heart. Descending aorta was cut, and the heart was flushed by injection of 7 mL EDTA buffer into the right ventricle. Ascending aorta was clamped by Reynolds forceps, and the heart was transferred to a 10 cm dish containing fresh EDTA buffer. Digestion was achieved by sequential injection of 10 mL EDTA buffer, 3 mL perfusion buffer, and 35 mL collagenase buffer into the LV. Constituent chambers (atria, LV, and right ventricle) were then separated, and the LV was gently pulled into 1 mm pieces using forceps. Cellular dissociation was completed by gentle trituration, and enzyme activity was inhibited by addition of 5 mL stop buffer (130 mM NaCl, 5mM KCl, 0.5 mM NaH_2_PO_4_, 10 mM HEPES, 10 mM glucose, 10 mM BDM,10 mM taurine, 1 mM MgCl_2_, 5% FBS). Cell suspension was passed through a 100 μm filter, and cells underwent 4 sequential rounds of gravity settling, using 3 intermediate calcium reintroduction buffers to gradually restore calcium concentration to physiological levels. The cell pellet in each round was enriched with myocytes and ultimately formed a highly pure myocyte fraction. Cardiac myocyte yields and percentage of viable rod-shaped cells were quantified using a hemocytometer. The cardiac myocytes were resuspended in prewarmed plating media and plated at an application-dependent density onto laminin-precoated(5 μg/mL) tissue culture plastic, in a humidified tissue culture incubator (37°C, 5% CO_2_). After 1 hour, and every 48 hours thereafter, media were changed to fresh, prewarmed culture media.

### Cardiac myocyte hypoxia and H_2_O_2_ injury models.

In this experiment, cardiac myocytes were cultured in a serum-and glucose-free medium for 24 hours for cardiomyocyte synchronization. Hypoxia injury was induced by placing the cells in a hypoxia chamber filled with 5% CO_2_ and 95% N_2_ at 37°C for 24 hours (14, 22). In H_2_O_2_ injury model, the injury induced by oxygen-free radicals in a serum-and glucose-free medium was treated with H_2_O_2_ (50 μM) for 12 hours (23).

### Oligo transfection and adenovirus-mediated gene transfer in vitro.

Oligo transfection was performed according to an established protocol (24). For the gene knockdown, *Morrbid* or serpine1 siRNA or Ad-*Morrbid* siRNA was added to the culture medium at a final oligonucleotide concentration of 50 nM or 30 MOI. Ad-*Morrbid* and control Ad-GFP was added with 30 MOI in a serum-free medium for 6–12 hours and was then changed to the normal medium.

### qPCR.

RNA expression in cultured cardiac cells and in mouse hearts was determined by qPCR as described (19). In brief, qPCR was performed on cDNA generated from 1 μg of total RNA using the protocol of the Luminaris HiGreen qPCR Master Mix (Thermo Fisher Scientific). Amplification and detection of specific products were performed with a QuantStudio3 System (Thermo Fisher Scientific). As an internal control, 18s was used for template normalization. The primers used were provided by Integrated DNA Technologies. Fluorescence signals were normalized to an internal reference, and the threshold cycle (C_t_) was set within the exponential phase of the PCR. The relative gene expression was calculated by comparing cycle times for each target PCR. The target PCR C_t_ values were normalized by subtracting the 18s C_t_ value, providing the ΔC_t_ value. The relative expression level between treatments was then calculated using the following equation: relative gene expression = 2^–(ΔCt^ ^sample^^–^^ΔCt^ ^control)^.

### Western blot analysis.

Proteins isolated from cultured cardiac myocytes and mouse hearts were determined by Western blot analysis. Equal amounts of protein were subjected to SDS-PAGE. A standard Western blot analysis was conducted using serpine1 antibody (1:1,000 dilution; ab182973, Abcam). β-Tubulin antibody (1:2,000 dilution; 2128S, Cell Signaling Technology) was used as the loading control. A cooling charged coupled device (CCD) imaging apparatus (Tanon-5200) was used for image capture.

### RIP assay.

The HCMs or NMCM specimens were lysed in ice-cold lysis buffer, and RIP was performed as previously described (25). Anti-serpine1 antibodies (catalog ab182973) were obtained from Abcam. The RIP assays were conducted using the Magna RIP RNA-Binding Protein Immunoprecipitation Kit (MilliporeSima) according to the manufacturer’s instructions.

### Statistics.

All data were checked for the normality assumption using the Shapiro-Wilk test, and the equal variance assumption was checked using the F test (for data with 2 groups) or Bartlett’s test (for data with 3 and more groups). Data satisfied these assumptions were analyzed using the unpaired 2-tailed Student’s *t* test (for 2 groups comparison) or 1-way ANOVA (for 3 or more groups comparison) when appropriate. Otherwise, they were examined using the nonparametric Mann-Whitney *U* or Kruskal-Walls tests. Values of data are presented as mean ± SEM, and the *P* value (including the adjusted *P* value) of 0.05 were considered as the significance threshold. All analyses were performed using the software GraphPad Prism8.0.

### Study approval.

All animal studies were approved by the IACUC at Southwest Medical University and were consistent with the *Guide for the Care and Use of Laboratory Animals* (National Academies Press, 2011).

### Data availability.

All data are available in this manuscript and in Supporting Data Values. All underlying data generated in this study are available from the corresponding author upon request.

## Author contributions

CZ was the PI of this study and conceived the concept, designed the experiments, and wrote the manuscript. Yang Yu was responsible for most of the animal studies. HY, QL, ND, JG, GQ, JF, Yajun Yu, and X Zhou performed molecular signaling studies, cellular functional studies, bioinformatics, histology, and animal model studies. JF, JW, X Zhang, and XW conducted KO experiments and adenovirus experiments.

## Supplementary Material

Supplemental data

Supporting data values

## Version 1 07/11/2023

In-Press Preview

## Version 2 08/22/2023

Electronic publication
