# Supplementary material for: Stress-enhanced cardiac lncRNA Morrbid protects hearts from acute myocardial infarction
Source: JCI Insight. 2023 Aug 22;8(16):e165568. doi: 10.1172/jci.insight.165568 (PMC10543728; doi:10.1172/jci.insight.165568)
Supplement: Supplemental data [file jciinsight-8-165568-s160.pdf]

**Supplementary Data**

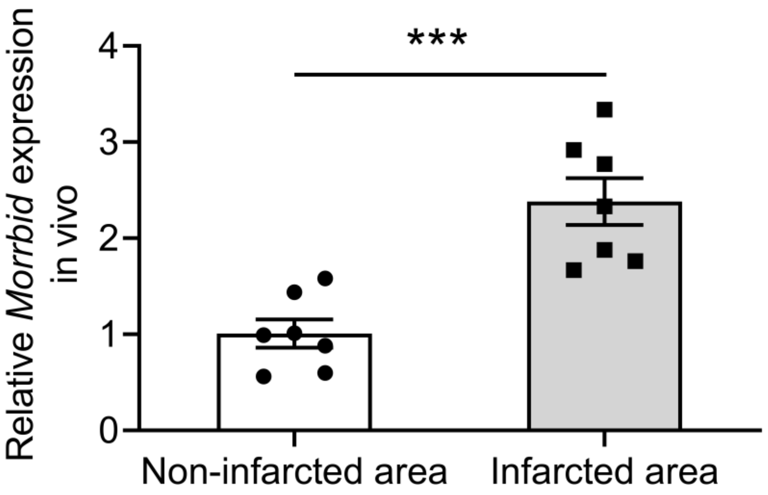


**Supplementary Figure 1.** The expression of Morrbid in non-infarcted area or infarcted area in mouse hearts at 24h after AMI (*P*<0.001; Non-infarcted area, n=7; Infarcted area, n=7). *** *P*<0.001.

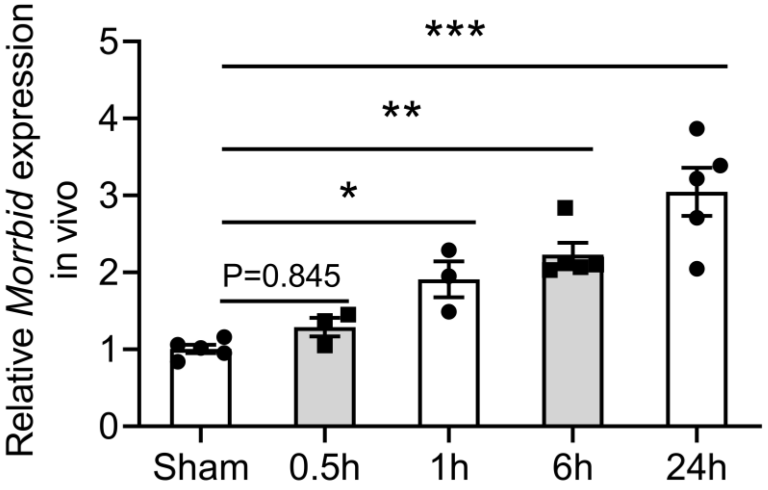


**Supplementary Figure 2.** The expression of Morrbid in sham-operated mouse hearts, and in mouse hearts at 0.5h, 1h, 6h and 24h after acute myocardial infarction (AMI) ($P_{0.5h}$=0.845; $P_{1h}$=0.039; $P_{6h}$=0.0012; $P_{24h}$<0.001; Sham, n=5; 0.5h, n=3; 1h, n=3, 6h, n=5; AMI, n=5). * P<0.05; ** P<0.01; *** P<0.001.

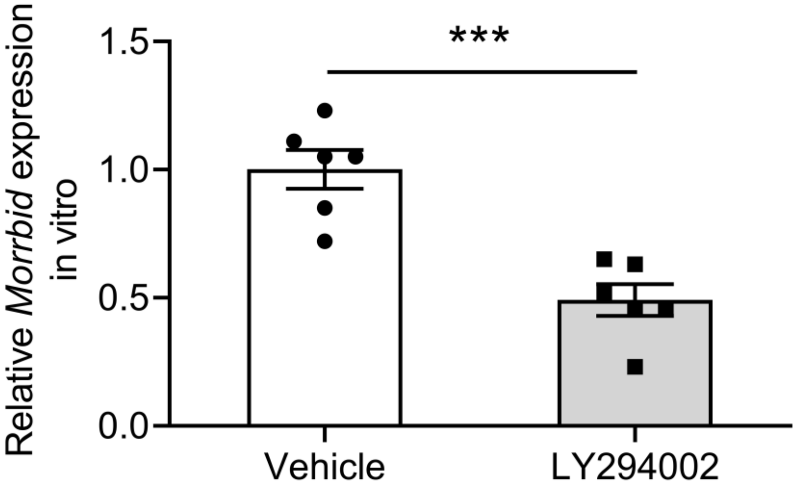


**Supplementary Figure 3.** The PI3-kinase inhibitor LY294002 inhibits the expression of Morrbid (*P*<0.001; Vehicle, n=6; LY294002, n=6) in cultured mouse cardiomyocytes. Note: *** *P*<0.001 by unpaired 2-tailed Student’s t tests.

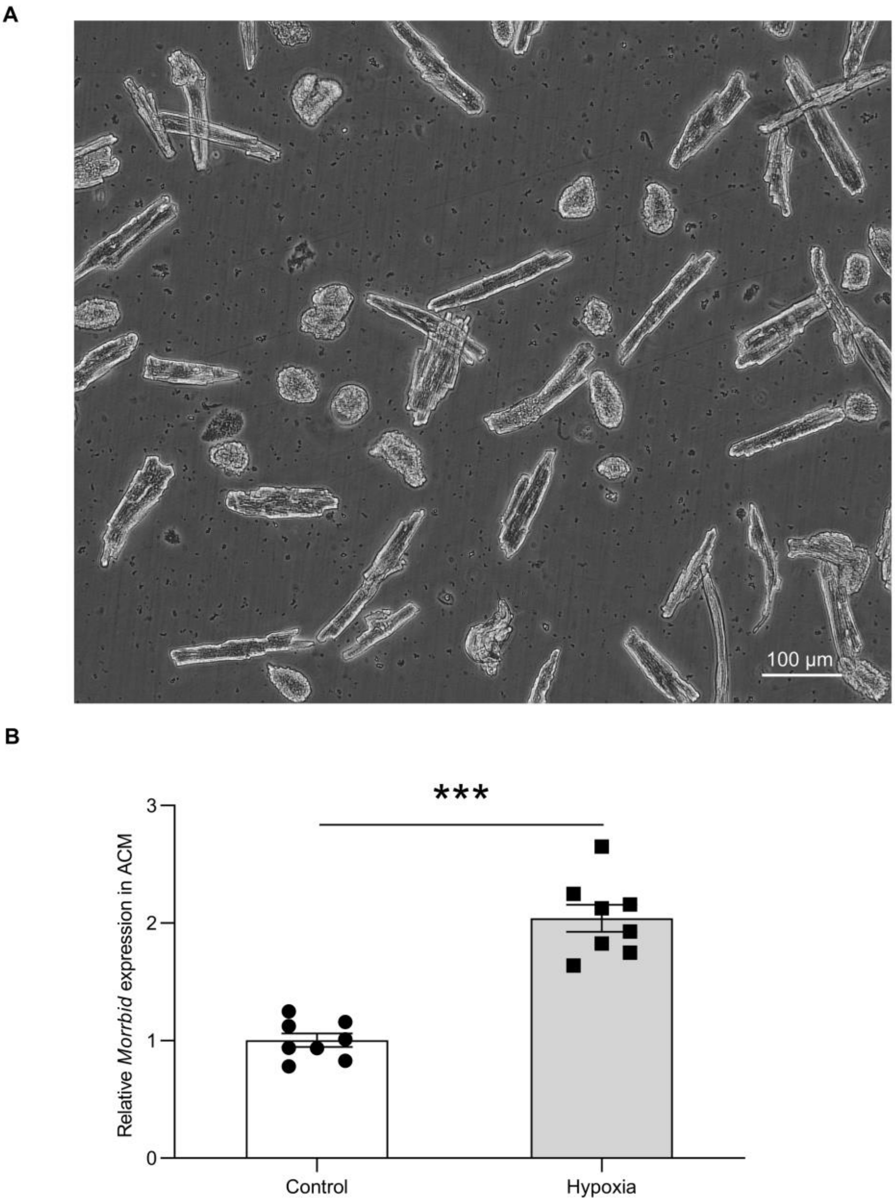


**Supplementary Figure 4. (A)** Representative photomicrographs from mouse adult cardiomyocytes (ACM). **(B)** The expression of Morrbid in mouse adult cardiomyocytes without hypoxia (Control group) and in mouse adult cardiomyocytes at 24h after hypoxia (P<0.001; Control, n=8; hypoxia, n=8). Note: *** P<0.001 by unpaired 2-tailed Student’s t tests.

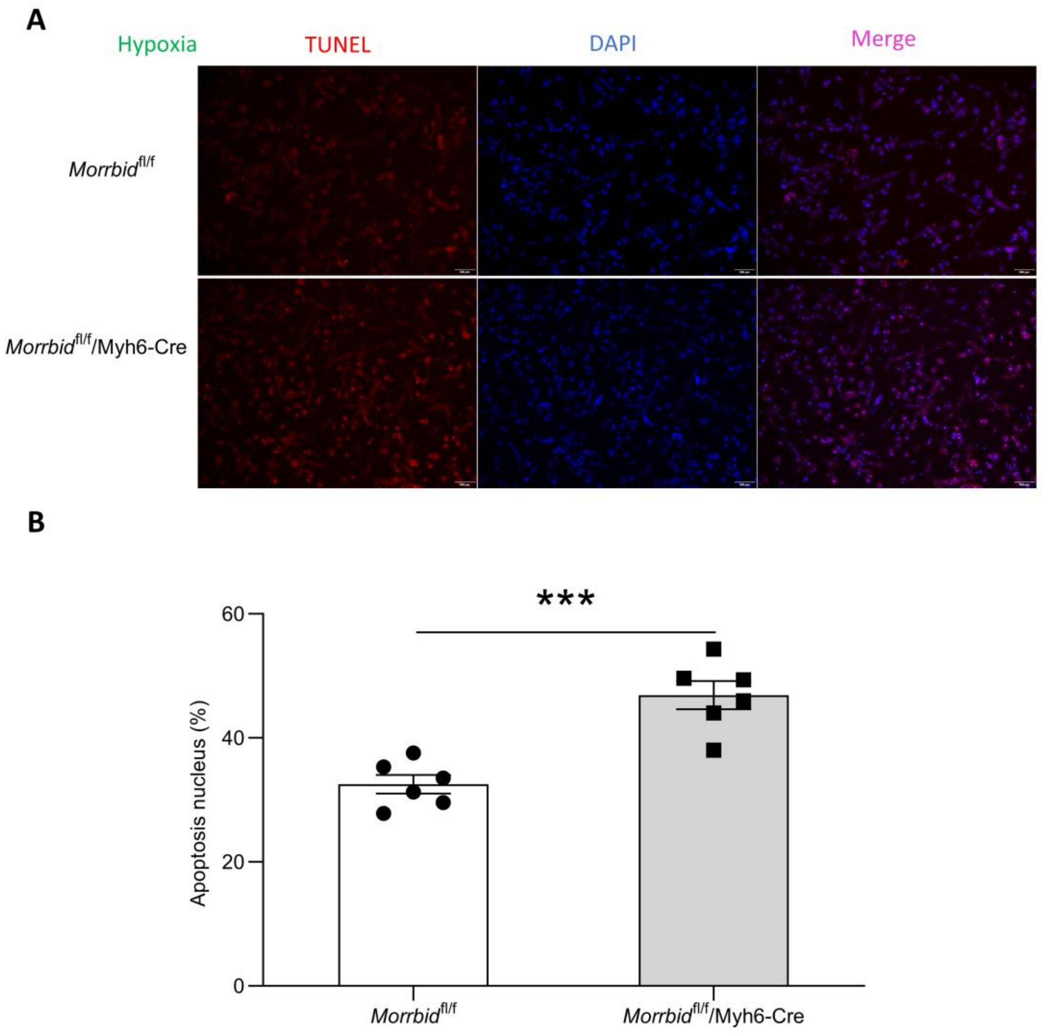


**Supplementary Figure 5.** Apoptosis is enhanced in adult cardiomyocytes from Morrbid knockout mice. Adult cardiomyocytes were isolated from Morrbid knockout mice (Morrbid$^{fl/fl}$/Myh6-Cre mice) and the control wild-type mice (Morrbid$^{fl/fl}$ mice). Cell apoptosis was induced by hypoxia for 24h in a serum free and low-glucose medium. Cell apoptosis (red color) was determined by TUNEL staining. **(A)** Representative TUNEL-stained photomicrographs of adult cardiomyocytes from Morrbid$^{fl/fl}$/Myh6-Cre mice or Morrbid$^{fl/fl}$ mice. **(B)** Quantitative analysis of the apoptotic nucleus (red color) in adult cardiomyocytes from Morrbid$^{fl/fl}$/Myh6-Cre mice or Morrbid$^{fl/fl}$ mice ($P<0.001$; Morrbid$^{fl/fl}$, n=6; Morrbid$^{fl/fl}$/Myh6-Cre, n=6). Note: *** $P<0.001$ by unpaired 2-tailed Student's t tests.

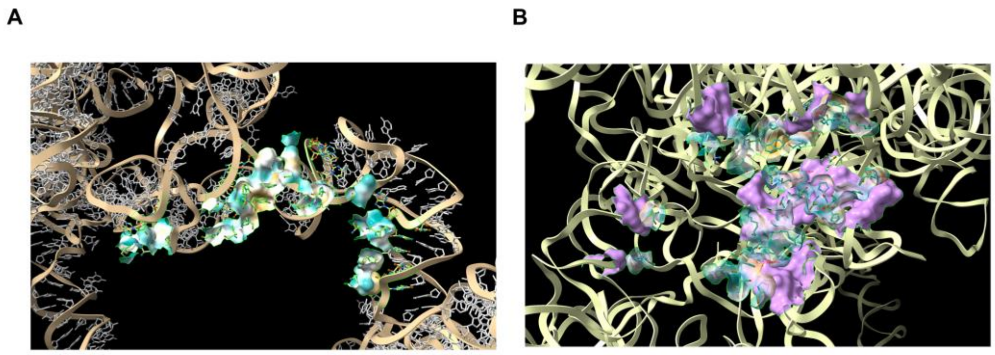


**Supplementary Figure 6. (A)** The detailed potential binding sites of human Morrbid with human serpine1. **(B)** The detailed potential binding sites of mouse Morrbid with mouse serpine1.

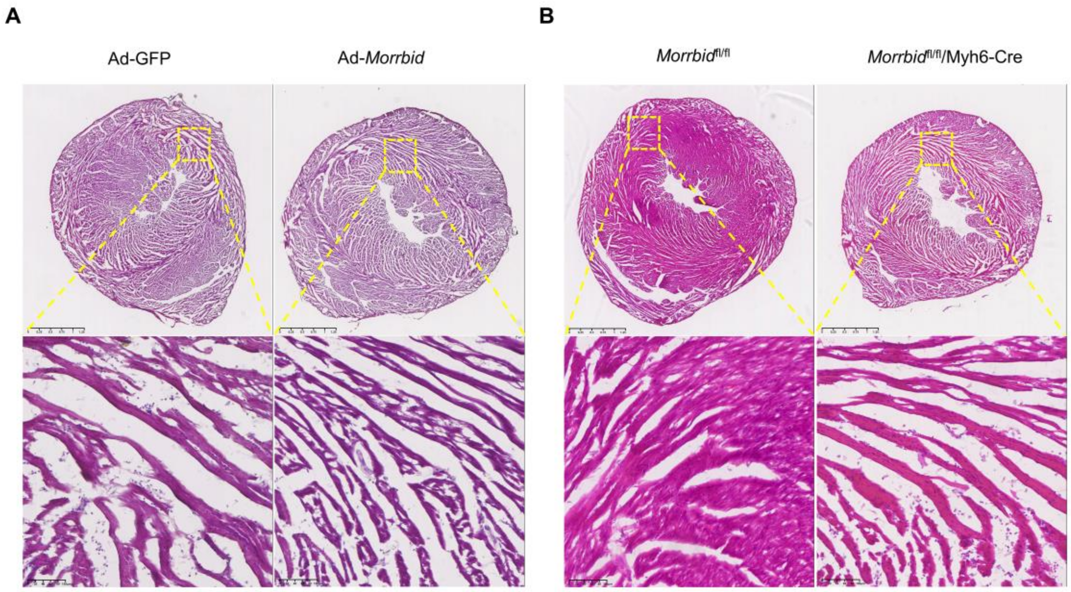


**Supplementary Figure 7.** Representative HE-stained photomicrographs of cardiac cells in heart sections from *Morrbid*$^{fl/fl}$/Myh6-Cre mice, *Morrbid*$^{fl/fl}$ mice and from wild type mice treated with Ad-GFP or Ad-*Morrbid* at 24 hours after AMI.

A

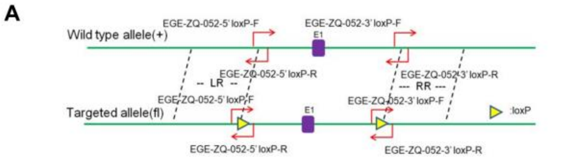


B

| Primer | Sequence (5'-3') | Tm (℃) | Product size (bp) |
|---|---|---|---|
| EGE-ZQ-052-5'loxP-F | GGGAGGGGTGGGCAAAGATATCAAC | 62 | WT:206 Mut:291 |
| EGE-ZQ-052-5'loxP-R | CGTTTCCATGCAAGAACCAAGAGCC | 62 | |
| EGE-ZQ-052-3'loxP-F | AACCACACTTCATGGGCCCTTCATT | 62 | WT: 261 Mut: 347 |
| EGE-ZQ-052-3'loxP-R | CAGCACCAAATCAAGTTGTGCCACC | 62 | |

| Primer | Sequence (5'-3') | Tm (℃) | Product size (bp) |
|---|---|---|---|
| 9543(Myh6-Cre) | ATGACAGACAGATCCCTCCTATCTCC | 62 | Mut = ~300 |
| 9544(Myh6-Cre) | CTCATCACTCGTTGCATCATCGAC | 60 | |
| oIMR8744(Myh6-Cre) | CAAATGTTGCTTGTCTGGTG | 58 | ~200 |
| oIMR8745(Myh6-Cre) | GTCAGTCGAGTGCACAGTTT | 60 | |

C

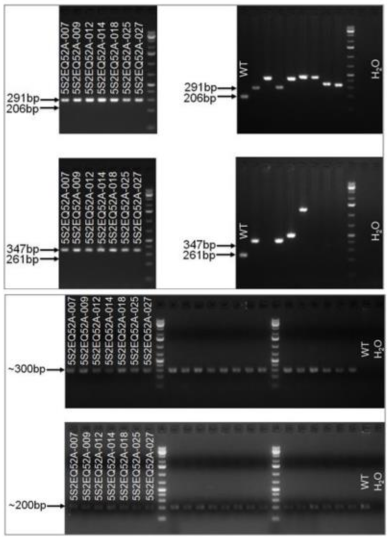


**Supplementary Figure 8. (A)** The targeting construct consisting of 1.3 kb arms of homologous genomic sequence immediately upstream (5’) of exon 1 and Intron 1 flanked by two loxP sites. **(B)** Design of primers for *Morrbid*$^{fl/fl}$ wild type Morrbid and Myh6-Cre. **(C)** PCR for verification of the Morrbid knockout.

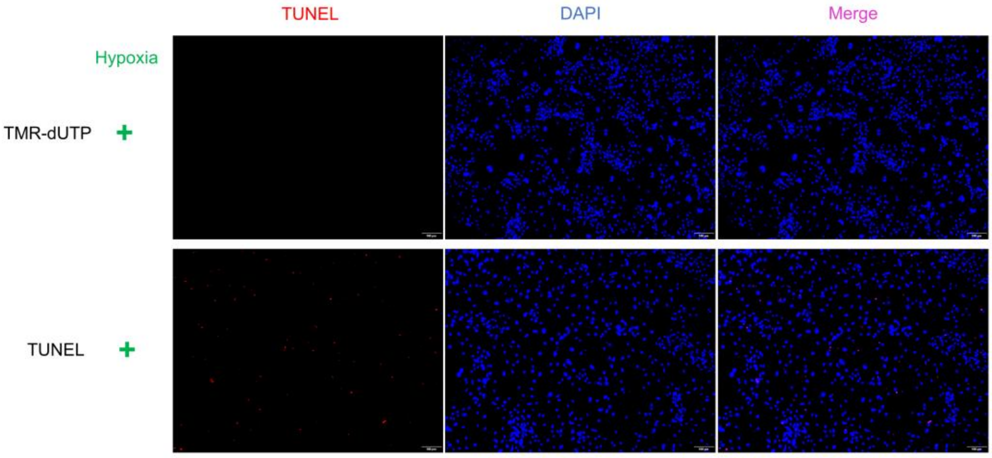


**Supplementary Figure 9.** Representative TUNEL-stained photomicrographs from cardiomyocytes treated with TMR-dUTP as negative control staining or normal TUNEL staining. Cultured cardiomyocyte apoptosis was induced by hypoxia for 24h in a serum free and low-glucose medium.

## Supplementary Table 1. Primers used in this study

| Primer name | Orientation | Primer sequences | Tm (℃) | Size of the amplicon in bp | GenBank Accession numbers |
|---|---|---|---|---|---|
| Mouse 18s | Forward | 5’-GTTCTTAGTTGGTGGAGCGATTT-3’ | 59.3 | 167 | BK000964 |
| | Reverse | 5’-AGGGCATCACAGACCTGTTATTG-3’ | 60.6 | | |
| Human 18s | Forward | 5’-CGGCTACCACATCCAAGGAA-3’ | 59.8 | 187 | AL353644 |
| | Reverse | 5’-GCTGGAATTACCGCGGCT-3’ | 60.5 | | |
| Mouse Serpine1 | Forward | 5’-TTCAGCCCTTGCTTGCCTC-3’ | 60.6 | 116 | AC147986 |
| | Reverse | 5’-ACACTTTTACTCCGAAGTCGGT-3’ | 59.6 | | |
| Human Serpine1 | Forward | 5’-GCACCACAGACGCGATCTT-3’ | 60.7 | 112 | AC004876 |
| | Reverse | 5’-ACCTCTGAAAAGTCCACTTGC-3’ | 58.4 | | |
| Mouse *Morrbid* | Forward | 5’-TCTGAGAATGAGGGGACTGG-3’ | 58.1 | 101 | AL844486 |
| | Reverse | 5’-TGTGCTGTGAAGATCCCAAG-3’ | 57.8 | | |
| Human *Morrbid* | Forward | 5’-ACTGGATGGTCGCTGCTTTT-3’ | 60.3 | 102 | AC017002 |
| | Reverse | 5’-CTTCCCAGGAACTGTGCTGT-3’ | 59.9 | | |
| EGE-ZQ-052-5’loxP | Forward | 5’-GGGAGGGGTGGGCAAAGATATCAAC-3’ | 65.2 | Mut:291 WT:206 | NC000068 |
| | Reverse | 5’-CGTTTCCATGCAAGAACCAAGAGCC-3’ | 65.0 | | |
| EGE-ZQ-052-3’loxP | Forward | 5’-AACCACACTTCATGGGCCCTTCATT-3’ | 65.0 | Mut:347 WT:261 | NC000068 |
| | Reverse | 5’-CAGCACCAAATCAAGTTGTGCCACC-3’ | 65.4 | | |
| 9543(Myh6-Cre) | Forward | 5’-ATGACAGACAGATCCCTCCTATCTCC-3’ | 62.7 | 300 | NC000080.7 |
| | Reverse | 5’-CTCATCACTCGTTGCATCATCGAC-3’ | 62.1 | | |
| oIMR8744(Myh6-Cre) | Forward | 5’-CAAATGTTGCTTGTCTGGTG-3’ | 55.8 | 200 | NC000080.7 |
| | Reverse | 5’-GTCAGTCGAGTGCACAGTTT-3’ | 58.4 | | |
